# Health Literacy Levels of Women Attending a Perinatology Outpatient Clinic for High-Risk Pregnancy Follow-Up

**DOI:** 10.7759/cureus.68267

**Published:** 2024-08-31

**Authors:** Mehmet Albayrak, Hilmi Furkan Arslan

**Affiliations:** 1 Obstetrics and Gynecology, Giresun Training and Research Hospital, Giresun, TUR; 2 Clinical Biochemistry, Giresun Obstetrics and Gynecology Education and Research Hospital, Giresun, TUR

**Keywords:** education, maternal health, socioeconomic status, pregnancy, health literacy

## Abstract

Background: Health literacy, defined as the ability to obtain, understand, evaluate, and apply health information with knowledge, motivation, and skills, is crucial for maintaining and improving quality of life. Despite the availability of health information, limited health literacy is linked to health disparities, inadequate self-management of chronic diseases, and poorer health outcomes.

Objective: The purpose of this study is to assess the health literacy of pregnant women who visit the Perinatology Outpatient Clinic for follow-up care. It seeks to identify gaps in knowledge and understanding that may impede effective healthcare delivery and inform targeted health education and public awareness programs to enhance health literacy.

Methods: This prospective survey study included 210 pregnant women aged 18 to 40 years attending the Perinatology Outpatient Clinic at Giresun Obstetrics and Gynecology Training and Research Hospital, Turkey. Participants completed a questionnaire on health literacy, sociodemographics, and basic health status via Google Forms (Google Inc., Mountain View, CA, USA). Statistical analysis was performed using SPSS Statistics version 26.0 (IBM Corp., Armonk, NY, USA), employing tests such as Kolmogorov-Smirnov, Mann-Whitney U, Kruskal-Wallis, Student’s t-test, ANOVA, Spearman, and Pearson correlation, and multivariate linear regression analysis.

Results: The mean age of participants was 29.97±5.44 years, with a mean health literacy score of 29.89±7.05. Education level and living place significantly influenced health literacy scores, with higher scores among those with higher education and urban living (p = 0.014 and p = 0.038, respectively). Economic status also significantly impacted health literacy, with lower scores among those with poor economic status (p<0.001). Health literacy scores were higher among those receiving health information from healthcare professionals (p = 0.006) and lower among those finding medical information from doctors insufficient (p = 0.008).

Conclusion: Health literacy is significantly influenced by education level, living place, and economic status. The study emphasizes the necessity of focused health education initiatives, especially for individuals with lower educational attainment and those residing in rural regions. Improving health literacy via efficient communication from medical professionals can benefit expectant mothers and their unborn children by lowering medical expenses and improving health outcomes.

## Introduction

The capacity to obtain, understand, evaluate, and apply health information to daily decision-making to maintain or improve quality of life about health services, disease prevention, and health promotion is commonly referred to as health literacy. Even with the ease of access to health information, a sizable segment of the populace continues to participate in unhealthy habits including smoking, inadequate physical exercise, and bad diet. These risk factors are linked to long-term conditions such as diabetes, which causes more than 75% of fatalities globally. Since poor health literacy outcomes are linked to low health outcomes and insufficient self-management of chronic diseases, it is a major contributor to health disparities. Those with inadequate health literacy use preventive services less frequently, stay in hospitals longer, have worse outcomes from medical procedures, and visit the doctor more frequently than people with excellent health literacy [1-3].

Maternal and fetal health is an area where the importance of health literacy is particularly evident. Poor health outcomes, underutilization of healthcare services, and unhealthy behaviors have all been associated with low health literacy. During pregnancy, low health literacy can make it challenging for expectant mothers to understand prenatal care guidelines, recognize potential issues, and follow medical advice. These difficulties can be harmful to the health of both the mother and her unborn child [4,5].

This study aims to evaluate the health literacy levels of women attending the Perinatology Outpatient Clinic for pregnancy follow-up. By identifying the health literacy levels in this high-risk population, we can identify gaps in knowledge and understanding that may impede effective healthcare delivery. This information is crucial for designing targeted health education and public awareness programs to improve health literacy among patients and the broader community.

In this particular clinical environment, our study will offer insightful information about the present level of health literacy among expectant mothers. It will also inform healthcare providers and policymakers about the necessary interventions to address health literacy deficiencies, thereby promoting better health outcomes for mothers and their babies.

## Materials and methods

This cross-sectional survey study approved by the Giresun Education and Research Hospital Ethics Committee (approval no. 24.04.2024/060) was conducted with pregnant women who were attending the Perinatology Outpatient Clinic at Giresun Obstetrics and Gynecology Training and Research Hospital, Turkey, for pregnancy follow-up. The primary aim of the study was to assess the health literacy levels of female patients in the outpatient perinatology clinic and explore potential correlations between health literacy and various demographic and health-related factors, with a particular focus on the education levels of at-risk pregnant women.

To determine the appropriate sample size, power analysis was performed using the G.Power program (Heinrich-Heine-Universität Düsseldorf, Düsseldorf, NWA, DEU). When the power of the study was taken as 99%, the alpha value as 0.05, and the effect size as 0.25, a sample size of a minimum of 140 participants was calculated. For the evaluation of prenatal screening tests (double and triple tests), chemiluminescence immunoassay (CLIA) sandwich method measurements were performed using Siemens Immulite 2000XPi Immunoassay System (Siemens Healthineers, Erlangen, BY, DEU) in the biochemistry laboratory. Evaluations were performed with PRISCA 5 prenatal risk assessment software (Siemens Healthineers). The study included 210 volunteer pregnant women, aged 18 to 40 years, who were referred to the Perinatology Outpatient Clinic due to high-risk pregnancy outcomes. 

Inclusion and exclusion criteria

Participants who were informed about the study and provided written consent, had the mental capacity to comprehend the questionnaire, had at least a primary school education, and were aged between 18 and 40 years, were included in the study. Exclusion criteria included patients who were illiterate or did not consent to participate and those with mental or central nervous system pathology or malignancy.

Data collection methods

Data for the study were collected using a structured questionnaire administered through Google Forms (Google Inc., Mountain View, CA, USA). The questionnaire consisted of two main sections, namely the 'descriptive characteristics form' and the 'short-form health literacy instrument' (SF-HLI). The descriptive characteristics form gathered sociodemographic and pregnancy-related information such as age, education level, employment status, income level, family type, place of residence, gestational age, whether the pregnancy was planned, sufficiency of information received during pregnancy, sources of health information, number of pregnancies, pregnancy intervals, and any high-risk conditions during previous pregnancies.

The SF-HLI, originally developed by Duong et al. in 2019, was used to measure health literacy. This instrument includes 12 items, each rated on a 4-point Likert scale ranging from 1 (very difficult) to 4 (very easy). The health literacy index was calculated using the formula: index = (mean - 1) / 3 × 50. The index value ranges from 0 to 50, with higher scores indicating better health literacy. Participants were asked to evaluate the difficulty of various tasks related to accessing and understanding health information, such as understanding medication leaflets, evaluating treatment options, and accessing mental health resources [6].

Statistical analysis

All statistical analyses were performed using SPSS Statistics version 26.0 (IBM Corp., Armonk, NY, USA). The Kolmogorov-Smirnov test was applied to evaluate the normality of data distribution. Categorical variables were represented through counts and percentages, while continuous variables were presented as either mean ± standard deviation (SD) or median (25th to 75th percentile), contingent on their distribution. The Mann-Whitney U test and the Kruskal-Wallis test were used to compare non-parametric variables, such as patient characteristics and health literacy. In contrast, parametric variables were compared using the Student's t-test and ANOVA. Post-hoc analyses were conducted with Tamhane's T2 test. The Pearson correlation test was used for groups with normally distributed data, whereas the Spearman correlation test was employed for non-parametric data groups. To identify independent variables related to patients' overall health literacy scores, a multivariate linear regression analysis was conducted. A p-value of less than 0.05 was considered statistically significant for all tests.

## Results

On analysis of the demographic data, the average age score of the 210 participants was found to be 29.97 years and 5.44 SD, respectively, and their health literacy score to be 29.89 and 7.05 SD, respectively. Based on educational attainment, health literacy scores varied statistically significantly (p = 0.014), with primary school graduates scoring worse than those with a high school or university degree. The place of residence had an impact on health literacy scores as well; people who lived in the city center scored substantially higher than people who lived in towns (p = 0.038). Table 1 shows a significant correlation (p<0.001) between lower health literacy scores and poor economic status.

**Table 1:** Patient characteristics and health literacy Non-parametric data are median (25th to 75th percentile), while parametric data are given as mean ± standard deviation. Each comparable superscript, i.e., ^'a' ^and ^'b'^ denotes a subset of group categories that do not differ statistically significantly from one another at the p = 0.05 level. *ANOVA test, **Student's t-test, ***Mann-Whitney U, ****Kruskal-Wallis test

Characteristics		N (%)	Mean ± SD or Median (25th to 75th percentile)	p-value
Education level	Primary	34 (17%)	26.71 ± 8.90^a^	0.014*
	High school	60 (30%)	30.39 ± 6.50^b^	
	Universty	106 (53%)	30.78 ± 6.40^b^	
Living place	City	94 (47%)	30.01 ± 7.91	0.038**
	Town	106 (53%)	26.74 ± 5.98	
Family type	Nuclear	186 (92%)	29. 17 (25.01 – 33.34)	0.094***
	Extended	16 (8%)	26.39 (18.79 – 33.98)	
Economic status	Bad	40 (20%)	26.39 (22.92 – 29.86)^a^	<0.001****
	Moderate	124 (62%)	29.17 (24.31 – 34.03)^b^	
	Good	36 (18%)	29.17 (22.92 – 35.42)^b^	
Pregnancy plan	Planned	144 (72%)	29.17 (25.01 – 33.34)	0.092***
	Unplanned	56 (28%)	27.78 (22.72 – 33.08)	
Considers the pregnancy information given to be sufficient	Sufficient	180 (90%)	30.19 ± 6.98	0.008**
	Insufficient	20 (10%)	24.16 ± 6.18	
Source of health information	Healthcare professionals	180 (90%)	29.17 (25.01 – 33.34)^a^	0.006****
	Internet/TV	34 (17%)	23.61 (22.72 – 28.62)^b^	
	Relations	16 (8%)	22.22 (15.82 – 26.39)^b^	
Number of pregnancy	First	82 (41%)	30.16 ± 6.31	0.087*
	2-4	106 (53%)	28.87 ± 7.47	
	>4	12 (6%)	29.42 ± 7.18	
Pregnancy interval	<24 months	74 (37%)	29.52 ± 6.37	0.163**
	>24 months	126 (63%)	30.68 ± 7.33	

There were no statistically significant differences in health literacy scores regarding family type (p = 0.094), pregnancy planning (p = 0.092), number of pregnancies (p = 0.087), or pregnancy interval (p = 0.163) (Table 2). Participants who received health information from healthcare professionals had significantly higher health literacy scores compared to those who obtained information from TV, the Internet, or relatives (p = 0.006). Conversely, participants who found the information provided by their doctors about pregnancy to be insufficient had significantly lower health literacy scores (p = 0.008) (Table 1). 

**Table 2:** Health literacy survey The data is shown as n (%).

Statements	Difficulty level	n (%)
Finding information about the treatment of diseases that concern you	Very difficult	4 (2%)
	Quite difficult	32 (16%)
	Quite easy	134 (67%)
	Very easy	30 (15%)
Understanding the leaflets for medications (drug information leaflets)	Very difficult	9 (4.5%)
	Quite difficult	22 (11%)
	Quite easy	133 (66.5%)
	Very easy	36 (18%)
Understanding the advantages and disadvantages of different treatment options for your diseases	Very difficult	7 (35%)
	Quite difficult	45 (22.5%)
	Quite easy	110 (55%)
	Very easy	38 (19%)
Calling an ambulance in case of an emergency	Very difficult	0 (0%)
	Quite difficult	0 (0%)
	Quite easy	118 (59%)
	Very easy	82 (41%)
Access to information on how to manage mental health problems such as stress or depression	Very difficult	4 (2%)
	Quite difficult	35 (17.5%)
	Quite easy	115 (57.5%)
	Very easy	46 (23%)
Understanding why you might need health screenings (such as breast exams, blood sugar tests, blood pressure checks)	Very difficult	0 (0%)
	Quite difficult	10 (5%)
	Quite easy	130 (65%)
	Very easy	60 (30%)
Deciding which vaccines you might need	Very difficult	12 (6%)
	Quite difficult	40 (20%)
	Quite easy	100 (50%)
	Very easy	48 (24%)
Deciding how to protect yourself from illnesses based on advice from friends and family	Very difficult	8 (4%)
	Quite difficult	47 (23.5%)
	Quite easy	108 (54%)
	Very easy	37 (18.5%)
Gaining information about activities that improve mental health (meditation, exercise, walking, pilates, etc.)	Very difficult	2 (1%)
	Quite difficult	9 (4.5%)
	Quite easy	133 (66.5%)
	Very easy	56 (28%)
Understanding information from the media (internet, newspapers, magazines) about how to be healthier	Very difficult	2 (1%)
	Quite difficult	25 (12.5%)
	Quite easy	125 (62.5%)
	Very easy	48 (24%)
Deciding which daily behaviors (drinking and eating habits, exercise, etc.) are related to your health	Very difficult	4 (2%)
	Quite difficult	12 (6%)
	Quite easy	128 (64%)
	Very easy	56 (28%)
Joining a sports club or exercise activity	Very difficult	23 (11.5%)
	Quite difficult	50 (25%)
	Quite easy	87 (43.5%)
	Very easy	40 (20%)

The results of the correlation analysis showed that the sources of health information, age, and place of residence were negatively connected with the total health literacy scores (r = -0.260, p<0.001; r = -0.199, p = 0.005; r = -0.260, p<0.001). Conversely, a positive association was discovered between health literacy scores, economic position, and education level (r = 0.191, p = 0.007; r = 0.204, p = 0.004). Table 3 shows that neither gestational age nor the adequacy of the information about pregnancy significantly correlated with health literacy scores.

**Table 3:** Correlation of the relationship between patient characteristics and health literacy score *Pearson correlation test, **Spearman correlation test

Characteristic	r-value	p-value
Age (years)	-0.199	0.005**
Pregnancy week	-0.033	0.645*
Education level	0.204	0.004*
Living place	-0.260	<0.001*
Economic status	0.191	0.007**
Considering pregnancy information given sufficient	-0.020	0.783*
Source of health information	-0.260	<0.001**

Possible risk variables connected to health literacy were found using a multivariable linear regression analysis. A substantial regression model that explains 37% of the variance in health literacy scores was found by this analysis (F(4, 189) = 13.22, p<0.001). The following independent factors (Table 4) were linked to health literacy: economic status (EXP(β) = 1.223, CI = 0.068 to 2.175), living place (EXP(β) = -1.184, CI = -2.967 to -0.021), education level (EXP(β) = 2.165, CI = 0.173 to 3.981), and source of health information (EXP(β) = -1.960, CI = -3.723 to -0.018).

**Table 4:** Multivariate linear regression analysis of variables associated with health literacy

Risk factor	Expected (β) value (95% CI)	p-value
Age (in years)	-0.044 (-0.072 to 0.029)	0.183
Education level (compared to primary education)	2.165 (0.173 to 3.981)	0.027
Living place (compared to city living)	-1.184 (-2.967 to -0.021)	0.019
Economic status (compared to poor economic status)	1.223 (0.068 to 2.175)	0.036
Source of health information	-1.960(-3.723 to -0.018)	0.368

## Discussion

This study highlights significant variations in health literacy levels among patients attending a perinatology outpatient clinic for high-risk pregnancy follow-up. The findings reveal that health literacy scores are influenced by various demographic factors, including education level, living place, and economic status. Specifically, higher education levels, urban living, and better economic status are associated with higher health literacy scores. These outcomes agree with earlier research indicating that socioeconomic status and educational attainment are crucial determinants of health literacy [7-10].

The strong correlation between health literacy and education level suggests that educational interventions could significantly enhance patients' ability to manage their health effectively. Programs designed to improve health literacy should focus on simplifying complex medical information and providing clear, accessible resources, particularly for those with lower educational levels and those living in rural areas. Furthermore, the higher health literacy scores for patients who received health information from healthcare professionals reinforce the role of healthcare providers as key information sources. Training for healthcare providers on effective communication strategies could further enhance patient understanding and outcomes [11,12].

Our study found a significant positive correlation between education level and health literacy scores, with higher education levels associated with better health literacy. This aligns with the findings of Svendsen et al., who demonstrated that individuals with higher socioeconomic positions, including education, exhibit better health literacy levels [7]. Schillinger emphasized that educational attainment is a significant social determinant of health, influencing health literacy and health outcomes [9]. Furthermore, Sheinis et al. and Chen et al. discovered a favorable correlation between knowledge of age-related pregnancy risks and greater health literacy scores, indicating the crucial role that education plays in promoting health literacy [13,14].

Our research indicates that living in an urban area is associated with higher health literacy scores than living in a town. This can be attributed to better availability and accessibility of health information and resources in urban areas. Sørensen et al. suggest that building health literacy system capacity is essential, particularly in rural areas where access to health information may be limited [1]. A large-scale study has highlighted that rural residents often face barriers to accessing health information, impacting their health literacy and health outcomes [15].

Economic status emerged as a significant factor, with lower economic status linked to poorer health literacy. This finding is consistent with Chu-Ko et al., who noted that socioeconomic disparities impact health literacy and related health behaviors [8]. Abel and McQueen highlighted that the COVID-19 crisis exacerbated existing health literacy inequalities, particularly among economically disadvantaged groups [16]. Furthermore, a study by Yee et al. reports that low health literacy is a risk factor for adverse pregnancy outcomes, particularly among economically disadvantaged populations [20].

Participants who relied on healthcare professionals for health information had significantly higher health literacy scores than those who relied on the Internet, TV, or relatives. This underscores the importance of credible sources in health literacy. Patil et al. found similar results in their study of US college students during the COVID-19 pandemic, emphasizing the need for reliable health information sources to improve health literacy [12]. A systematic review found that health literacy interventions focusing on credible sources significantly improved knowledge and pregnancy outcomes [17].

Our findings align with broader research indicating that health literacy is crucial for effective health management and improved health outcomes [18]. Higher health literacy levels among pregnant women are associated with better prenatal care and health behaviors. Tavananezhad et al. found a positive relationship between health literacy and empowerment in pregnant women, further supporting the critical role of health literacy in promoting maternal health [19]. Inadequate health literacy is also linked to negative maternal and neonatal outcomes, such as a higher risk of cesarean delivery, severe perineal tears, small-for-gestational-age status, low birth weight, and low five-minute Apgar scores [20].

Enhancing health literacy, particularly among vulnerable populations especially those with lower education levels or economic status, is crucial. Van Kessel et al. argue that digital health literacy should be considered a super determinant of health, and interventions should focus on improving access to and the quality of digital health information [21]. Integrating health literacy into prenatal care programs, as suggested by Nawabi et al., could significantly improve maternal health outcomes [22]. A systematic review by Zibellini et al. found that health literacy interventions improved knowledge, behaviors, and outcomes in pregnant women, emphasizing the need for targeted educational programs [23].

Study limitations

There are several limitations to this study. First, as it was conducted at a single center, the findings may not be widely generalizable. Second, the self-reported measures of health literacy could be affected by response bias. Additionally, the cross-sectional design of the study prevents causal inferences. Future research should employ longitudinal designs to better understand the changes in health literacy over time and its effects on pregnancy outcomes.

Future studies should explore the effectiveness of specific educational interventions in improving health literacy and patient outcomes in perinatology settings. Additionally, research should investigate the role of digital health literacy, especially in the context of increasing the use of digital health technologies. Digital health literacy is emerging as a critical component of overall health literacy, and its impact on maternal and fetal health warrants further exploration.

## Conclusions

This study emphasizes the crucial role of health literacy in managing high-risk pregnancies. The results point to the necessity of targeted interventions to enhance health literacy, especially for patients with lower education levels and those residing in rural areas. Improving health literacy can result in better health outcomes, lower healthcare costs, and enhanced overall well-being for pregnant women and their babies. Healthcare providers should receive training to communicate health information effectively, ensuring that all patients possess the knowledge and skills needed to make informed health decisions.
